# Supplementary figures and images for: Coupled equilibria of dimerization and lipid binding modulate SARS Cov 2 Orf9b interactions and interferon response
Source: eLife. 2025 Sep 17;14:RP106484. doi: 10.7554/eLife.106484 (PMC12443476; doi:10.7554/eLife.106484)

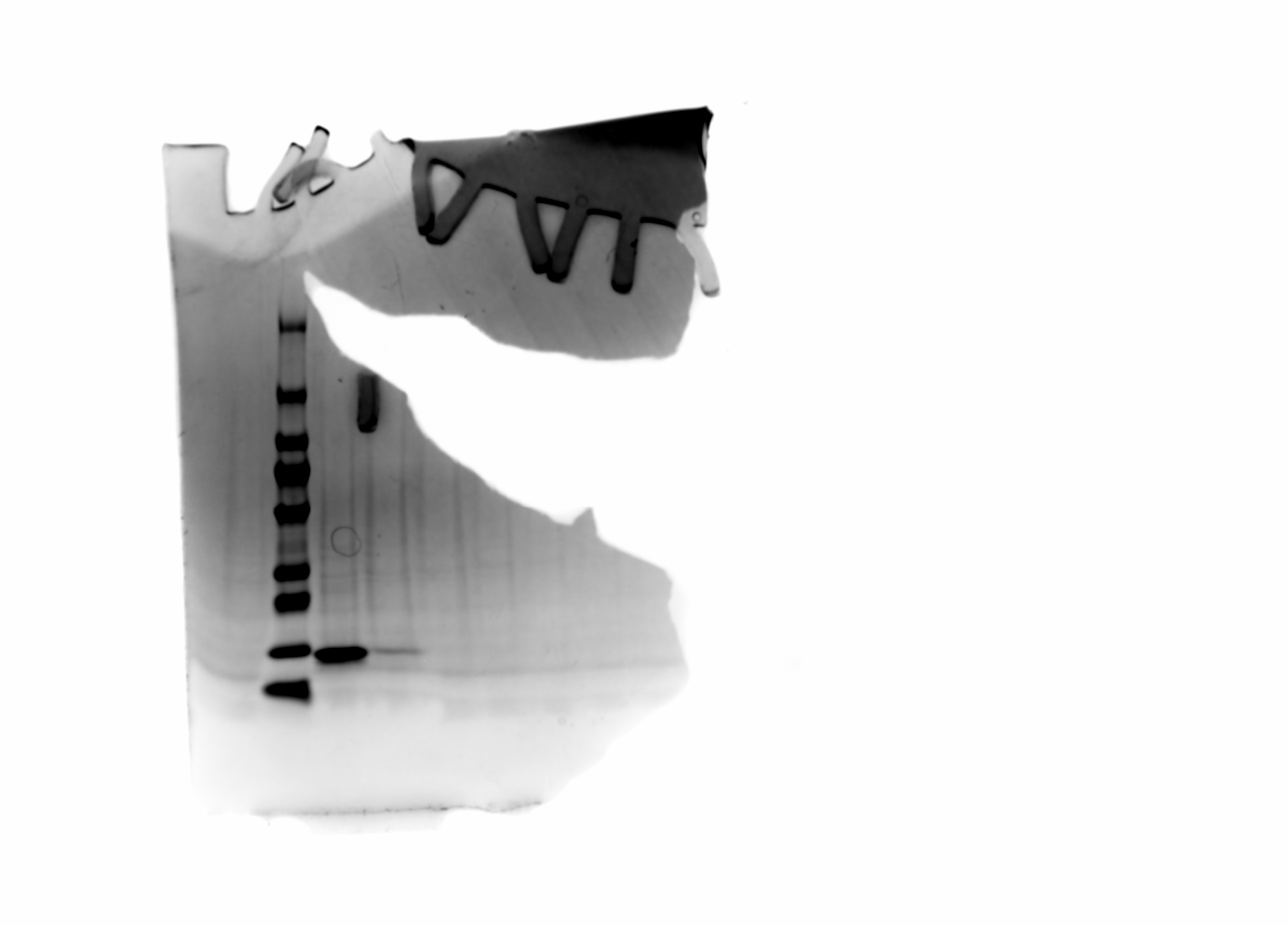

Supplement: Figure 3—source data 1. [file elife-106484-fig3-data1.zip › 2023-10-12_orf9b_dilution_s75_monomer_dimer_longstain.tif]

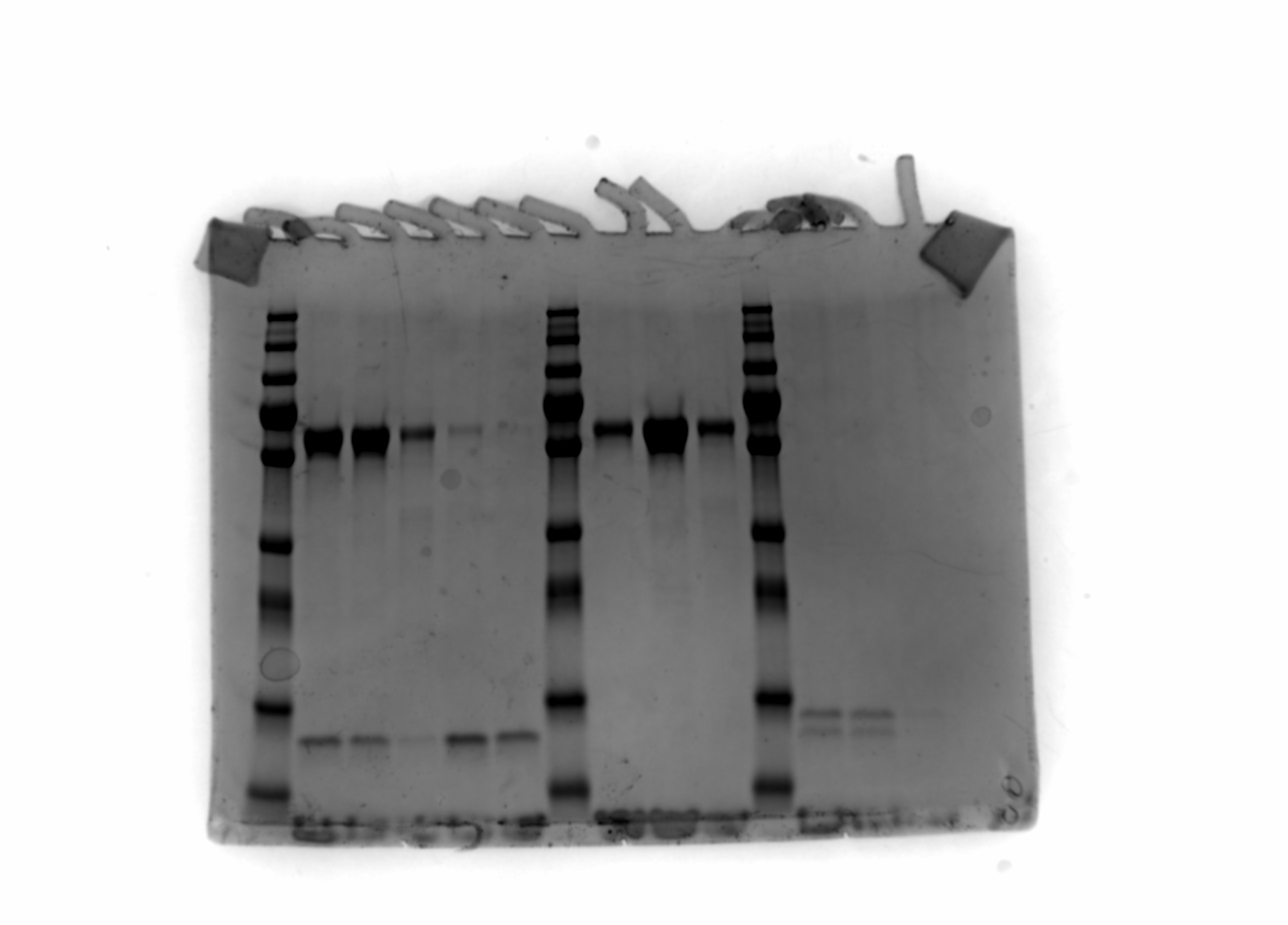

Supplement: Figure 3—figure supplement 1—source data 1. [file elife-106484-fig3-figsupp1-data1.zip › 2024-03-11_tom70-orf9b_tom70_orf9b.tif]

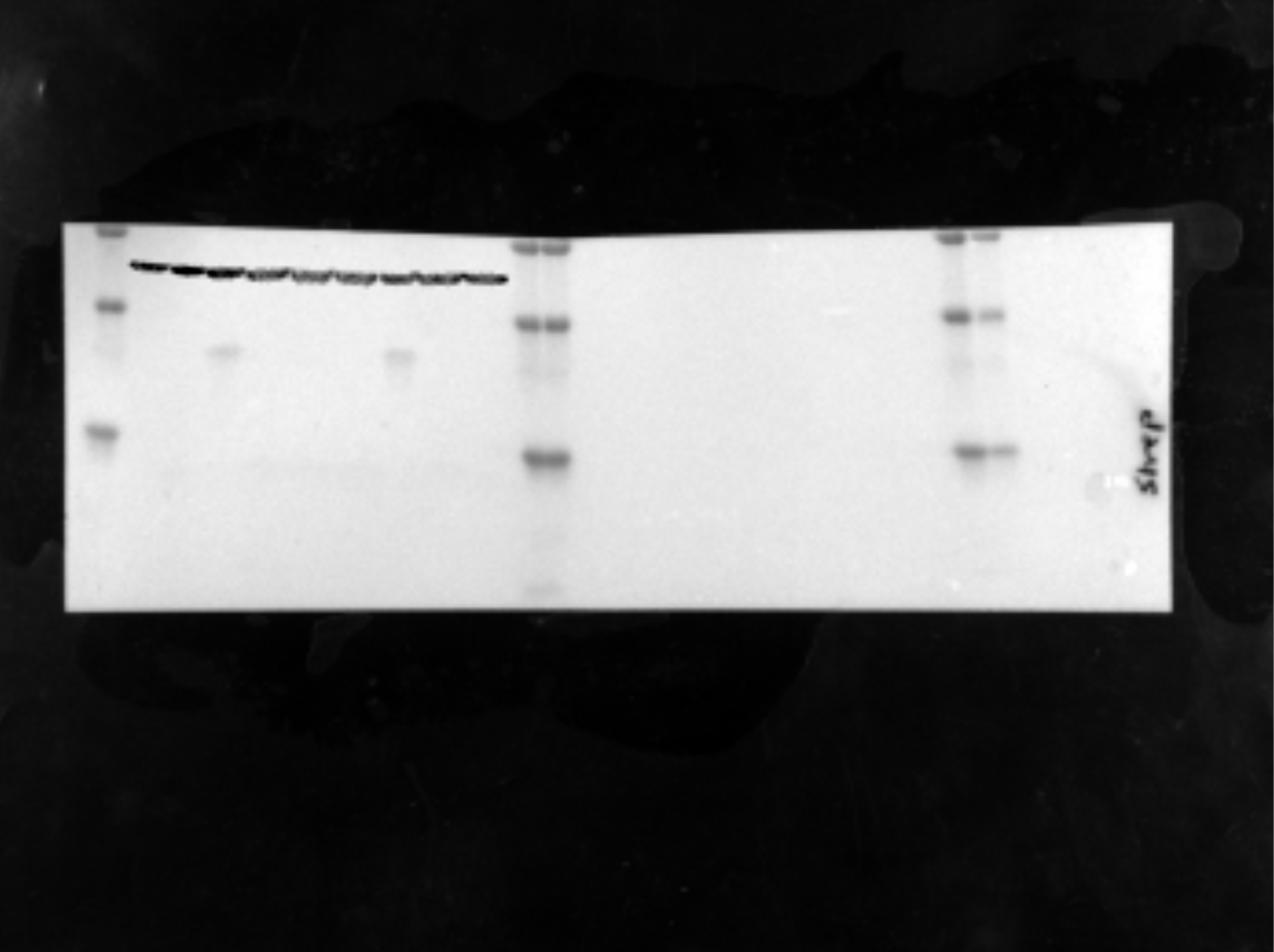

Supplement: Figure 5—source data 1. [file elife-106484-fig5-data1.zip › figure 5-source data/Actin.tif]

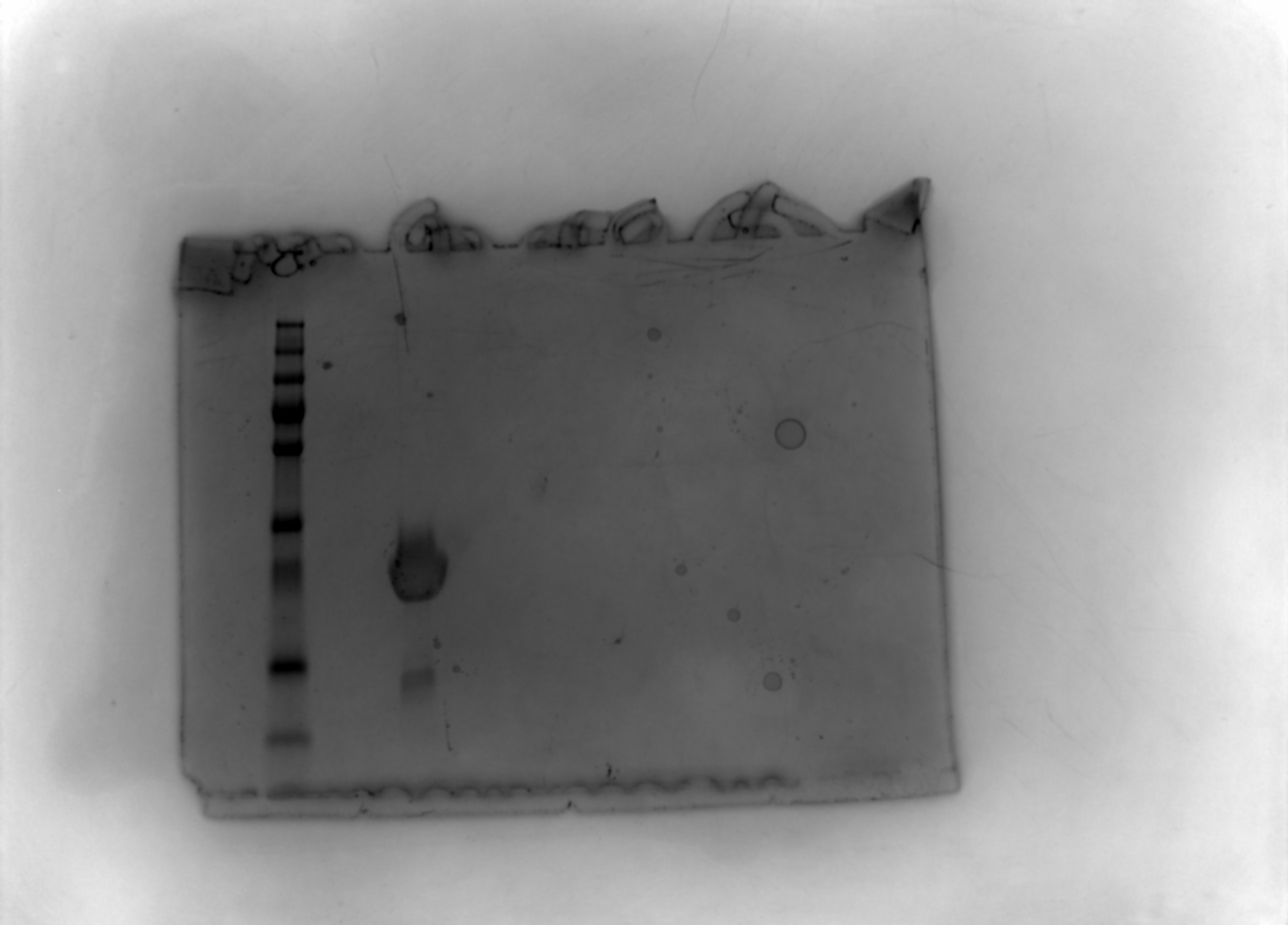

Supplement: Figure 5—figure supplement 1—source data 1. [file elife-106484-fig5-figsupp1-data1.zip › 2024-09-02_orf9b_4xSGG.tif]

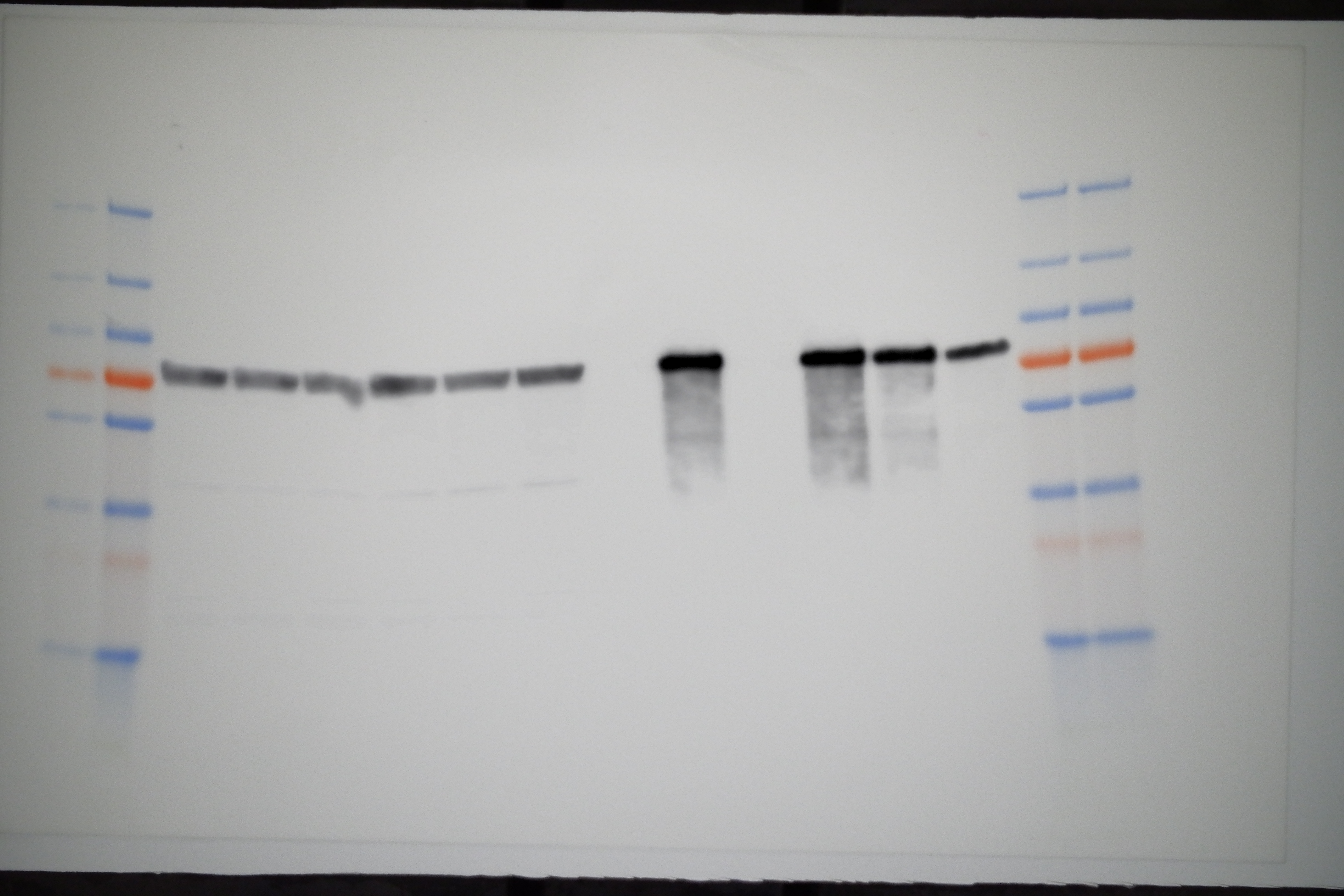

Supplement: Figure 6—source data 1. [file elife-106484-fig6-data1.zip › figure 6-source data/Tom70 WCL.tiff]

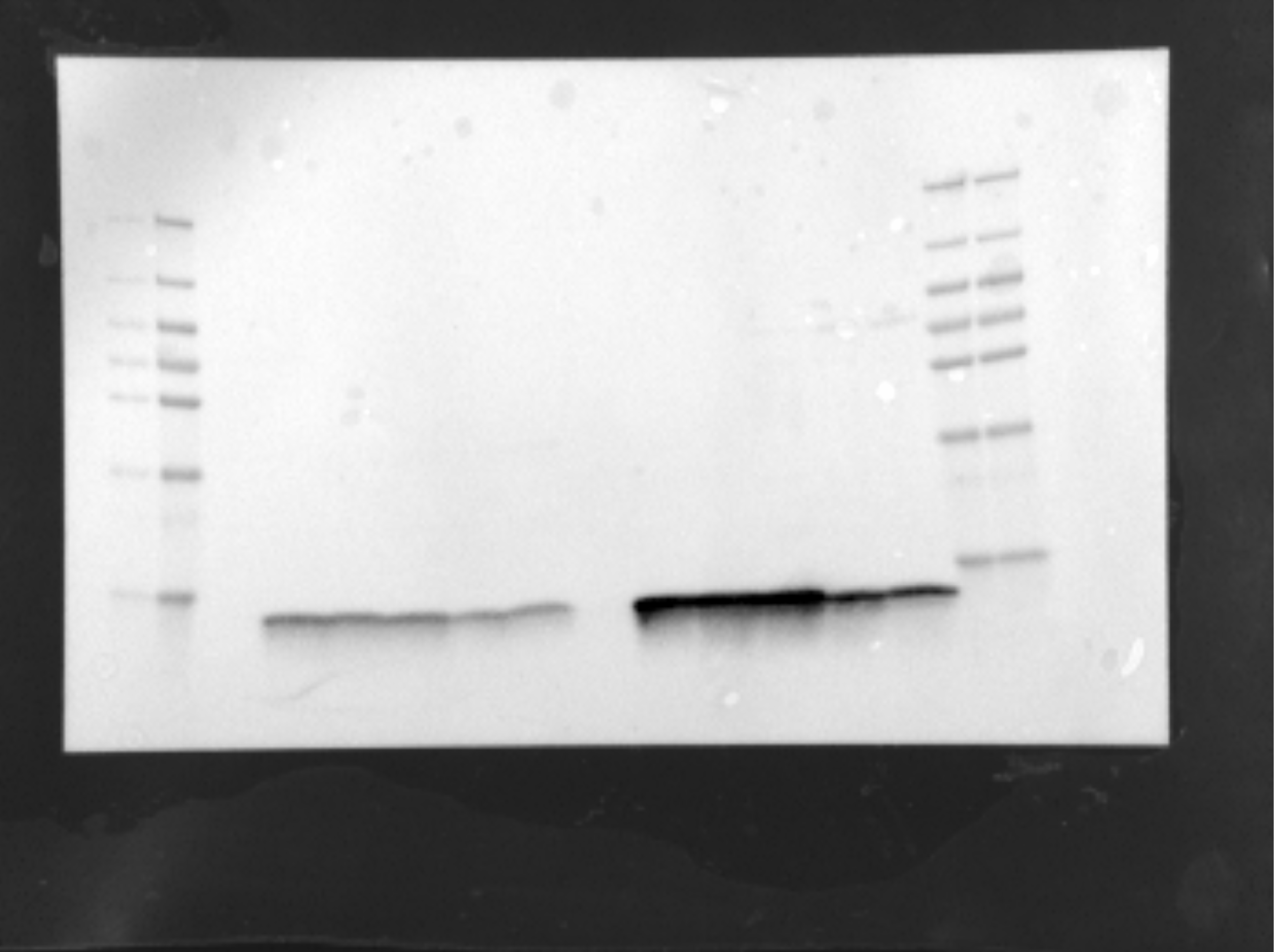

Supplement: Figure 6—source data 1. [file elife-106484-fig6-data1.zip › figure 6-source data/Strep-Orf9b IP.tif]

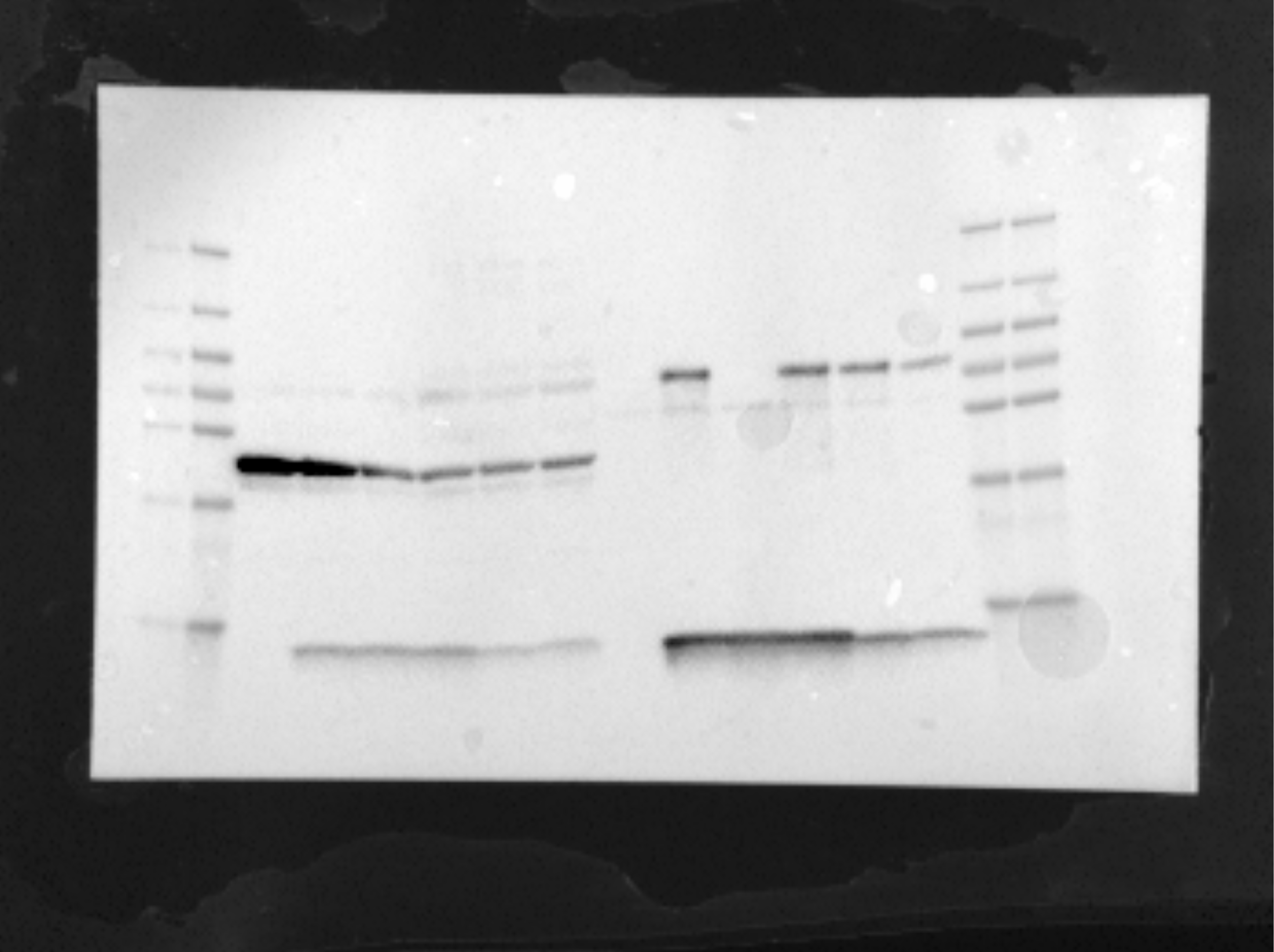

Supplement: Figure 6—source data 1. [file elife-106484-fig6-data1.zip › figure 6-source data/Actin.tif]

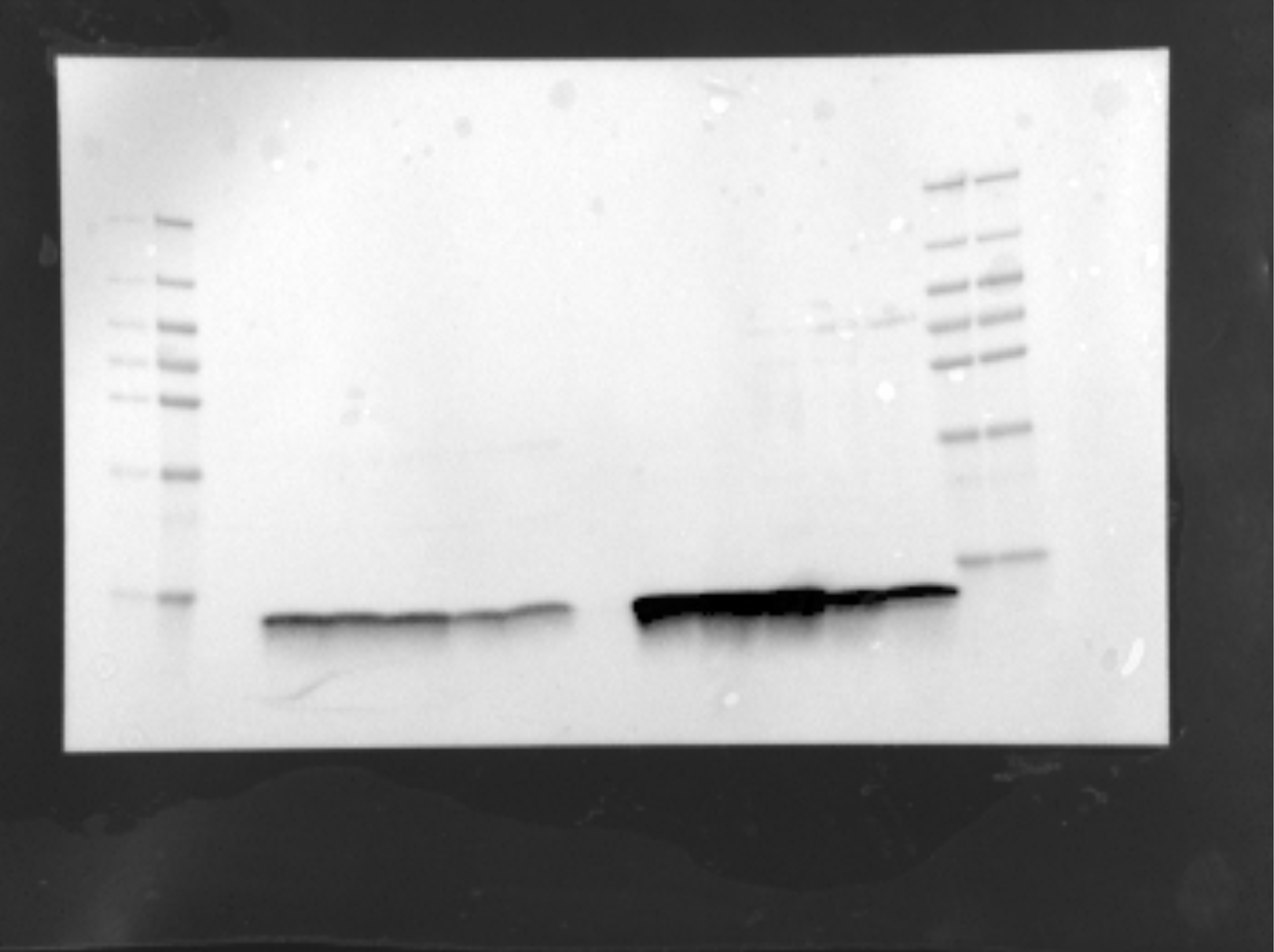

Supplement: Figure 6—source data 1. [file elife-106484-fig6-data1.zip › figure 6-source data/Strep-Orf9b WCL.tif]
