# Supplementary material for: Coupled equilibria of dimerization and lipid binding modulate SARS Cov 2 Orf9b interactions and interferon response
Source: eLife. 2025 Sep 17;14:RP106484. doi: 10.7554/eLife.106484 (PMC12443476; doi:10.7554/eLife.106484)
Supplement: Figure 3—source data 2. [file elife-106484-fig3-data2.zip › fig 3 source data 2.pdf]

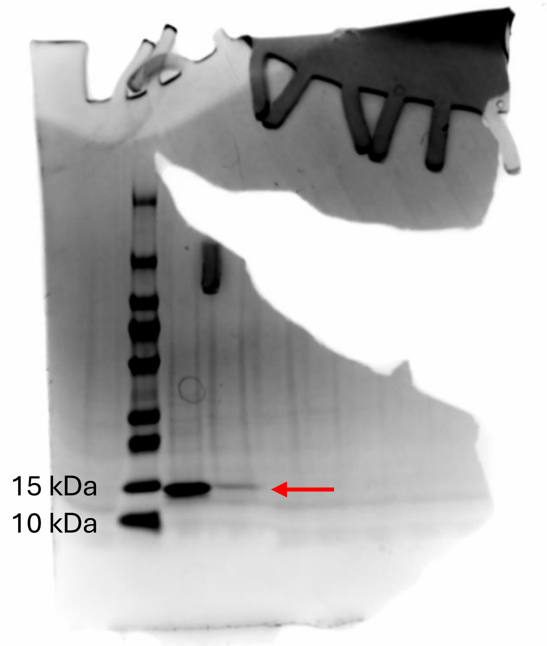

Figure 3 Source Data 2. Original SDS-PAGE corresponding to Figure 3 panel B. The left and right lanes correspond to samples taken from separate SEC peaks of Orf9b homodimer and monomer. Red arrow marks where the bands of interest are located. Molecular weights are listed based on the protein ladder. Gel was ripped during transfer to imager and did not contain any other loaded lanes.
