# Supplementary material for: Coupled equilibria of dimerization and lipid binding modulate SARS Cov 2 Orf9b interactions and interferon response
Source: eLife. 2025 Sep 17;14:RP106484. doi: 10.7554/eLife.106484 (PMC12443476; doi:10.7554/eLife.106484)
Supplement: Figure 3—figure supplement 1—source data 2. [file elife-106484-fig3-figsupp1-data2.zip › fig 3 sup 1 source data 2.pdf]

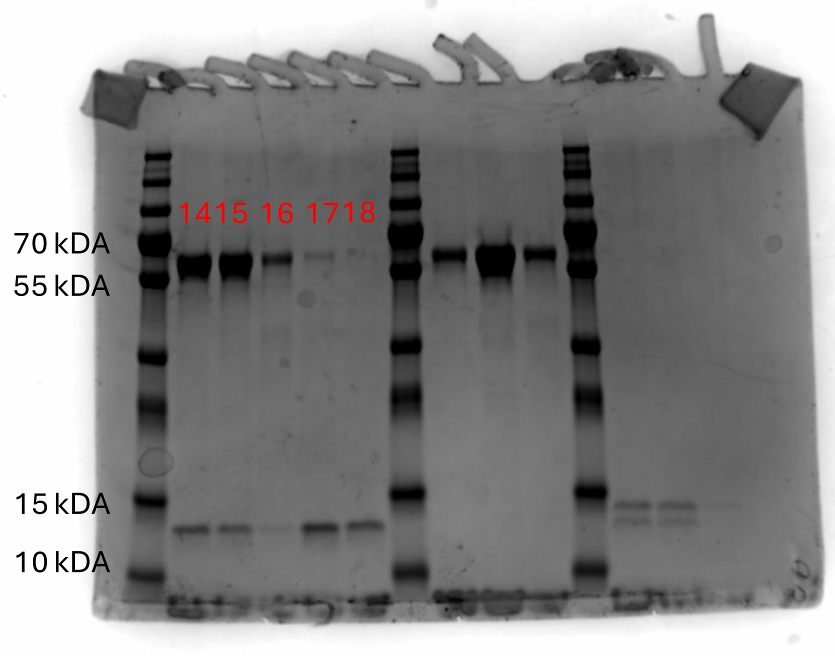

Figure 3 Supplemental 1 Source Data 2. Original SDS-PAGE corresponding to Figure 3 Supplemental 1 panel B. The left most lanes correspond to samples taken from SEC elution of Tom70:Orf9b. Red labeled numbers at the top correspond to fraction numbers taken from SEC chromatogram analysis software. Molecular weights are listed based on the protein ladder. Unlabeled middle and right lanes corresponded to a separate unrelated experiment that was run on the same gel.
