# Supplementary material for: Coupled equilibria of dimerization and lipid binding modulate SARS Cov 2 Orf9b interactions and interferon response
Source: eLife. 2025 Sep 17;14:RP106484. doi: 10.7554/eLife.106484 (PMC12443476; doi:10.7554/eLife.106484)
Supplement: Figure 5—source data 2. [file elife-106484-fig5-data2.zip › figure 5 source data 2.pdf]

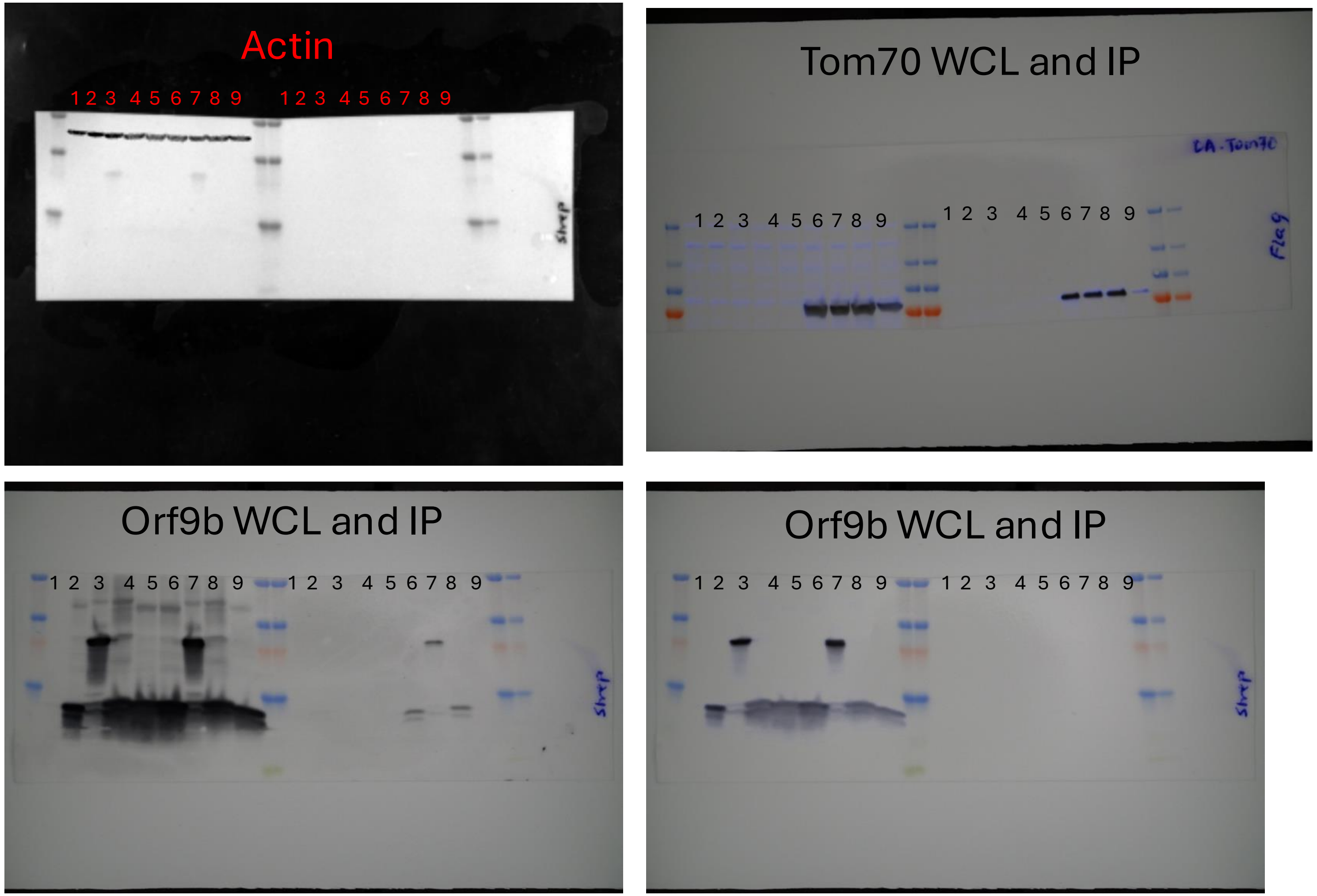

1: vector
2: -Orf9b WT
3: -Orf9b fused
4: -Orf9b truncated
5: -Orf9b S53/S50E
6: +Orf9b WT
7: +Orf9b fused
8: +Orf9b truncated
9: +Orf9b S53/S50E

Figure 5 Source Data 2. Original membranes corresponding to Figure 5 panel F. Top left are actin loading controls. Top right are flag tagged Tom70 lanes from whole cell lysate (left) and from immunoprecipitation (right). Bottom left are the strep tagged Orf9b lanes from whole cell lysate (left) and from immunoprecipitation (right). Bottom right is the same gel as bottom left but with lower exposure settings. Order of lanes is identical to the descriptions in Figure 5 panel B. Lanes are numbered 1-9 with a legend describing the conditions of each lane.
