# Supplementary material for: Coupled equilibria of dimerization and lipid binding modulate SARS Cov 2 Orf9b interactions and interferon response
Source: eLife. 2025 Sep 17;14:RP106484. doi: 10.7554/eLife.106484 (PMC12443476; doi:10.7554/eLife.106484)
Supplement: Figure 5—figure supplement 1—source data 2. [file elife-106484-fig5-figsupp1-data2.zip › fig 5 sup 1 source data 2.pdf]

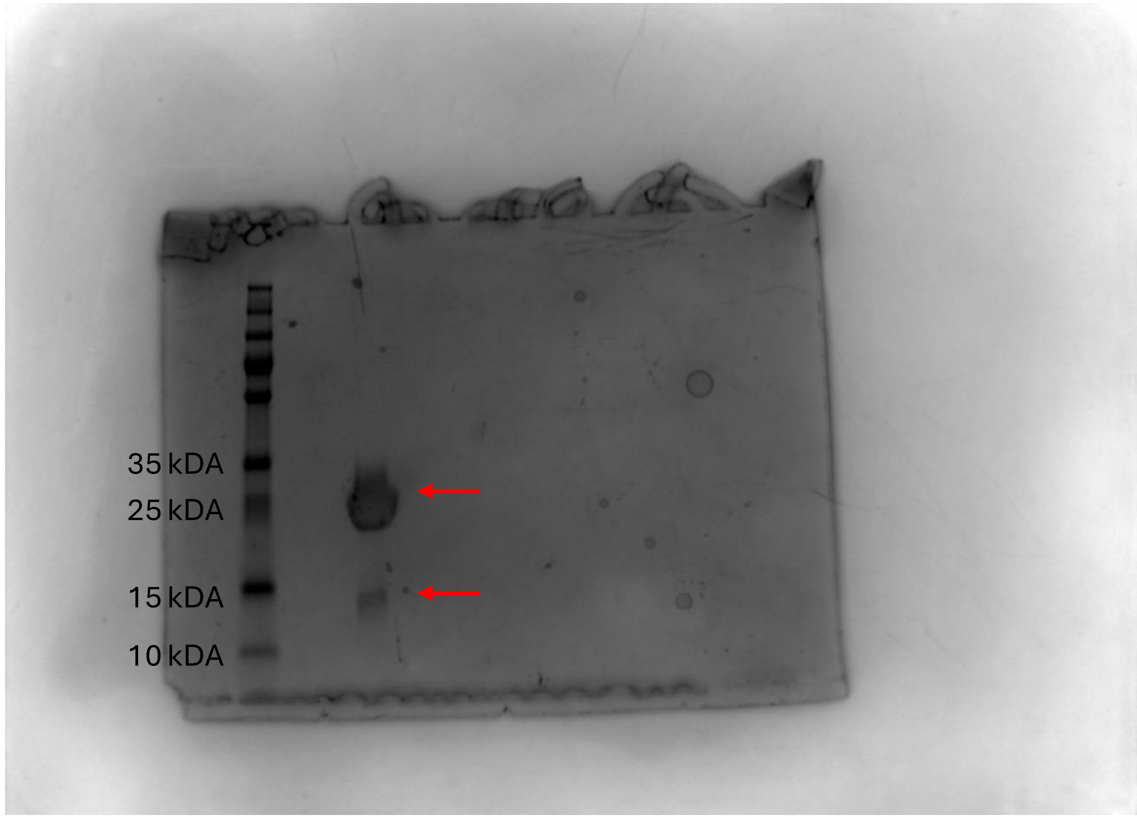

Figure 5 Supplemental 1 Source Data 2. Original SDS-PAGE corresponding to Figure 5 Supplemental 1 panel B. Red arrows indicate the bands corresponding to the Orf9b homodimer fusion product (top band) and the monomeric degradation product (lower band). Molecular weights are listed based on the protein ladder.
