# Supplementary material for: Coupled equilibria of dimerization and lipid binding modulate SARS Cov 2 Orf9b interactions and interferon response
Source: eLife. 2025 Sep 17;14:RP106484. doi: 10.7554/eLife.106484 (PMC12443476; doi:10.7554/eLife.106484)
Supplement: Figure 6—source data 2. [file elife-106484-fig6-data2.zip › figure 6 source data 2.pdf]

Actin

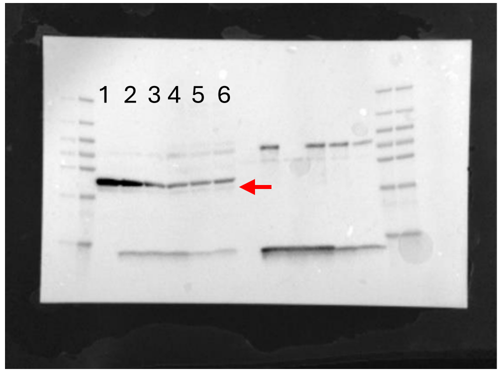

Tom70 WCL and IP

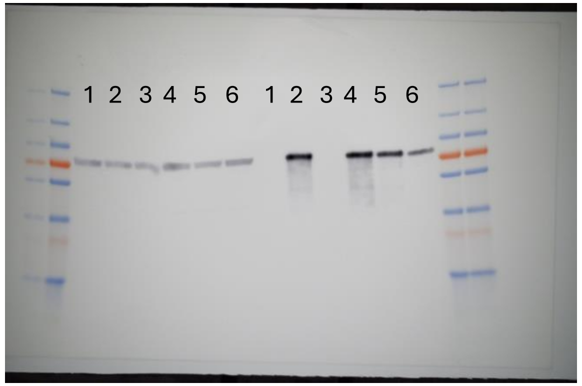

Orf9b WCL and IP

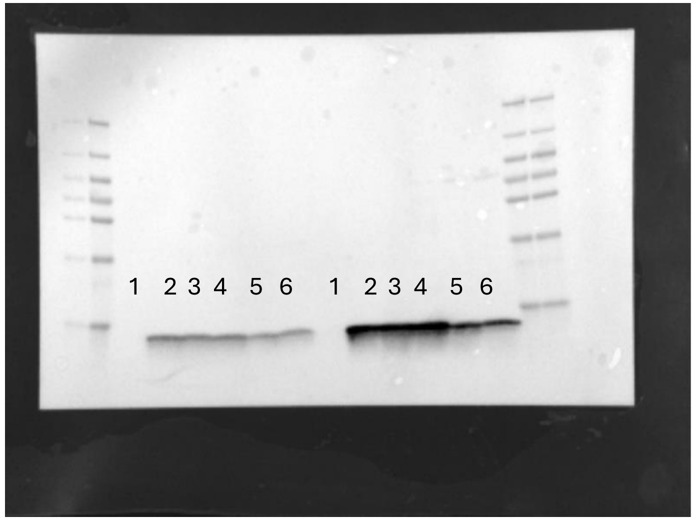

1: vector
2: WT Orf9b
3: S50/53E Orf9b
4: Lambda
5: Omicron
6: Delta

Figure 6 Source Data 2. Original membranes corresponding to Figure 6 panel E. Top left panel shows red arrow marking actin loading controls. Top right panel shows flag tagged Tom70 lanes from whole cell lysate (left) and from immunoprecipitation (right). Bottom left are the strep tagged Orf9b lanes from whole cell lysate (left) and from immunoprecipitation (right). Order of lanes is identical to the descriptions in Figure 5 panel B. Lanes are numbered 1-6 with a legend describing the conditions of each lane.
